# *Neisseria lactamica* Causing a Lung Cavity and Skin Rash in a Renal Transplant Patient: First Report from India

**DOI:** 10.1155/2016/1932963

**Published:** 2016-02-23

**Authors:** Khalid Hamid Changal, Adnan Raina, Sheikh Shoaib Altaf

**Affiliations:** ^1^Internal Medicine, Sher-i-Kashmir Institute of Medical Sciences, Srinagar 190011, India; ^2^Internal Medicine, Mercy Catholic Medical Center, Philadelphia, PA 19026, USA

## Abstract

*Neisseria lactamica*, a commensal, has been very rarely reported to cause diseases in immunocompromised hosts. In medical literature, there is only one report of a cavitatory lung lesion caused by it. The patient was a kidney transplant recipient.* Neisseria lactamica* was found to be the cause of his pulmonary cavity and a desquamating rash on feet. With the rapidly spreading medical advance, more and more patients are getting organ transplants, so the population of immunocompromised people is on the rise. We expect more sinister and less expected organisms to cause diseases in patients who have organ transplants.

## 1. Introduction

*Neisseria lactamica* is a normal commensal of the upper respiratory tract in humans. In immunocompromised hosts this organism may become pathogenic and cause diseases. There have been only a few reports of this organism causing a disease, mostly in immunocompromised hosts.

## 2. Case

**Figure 1:**
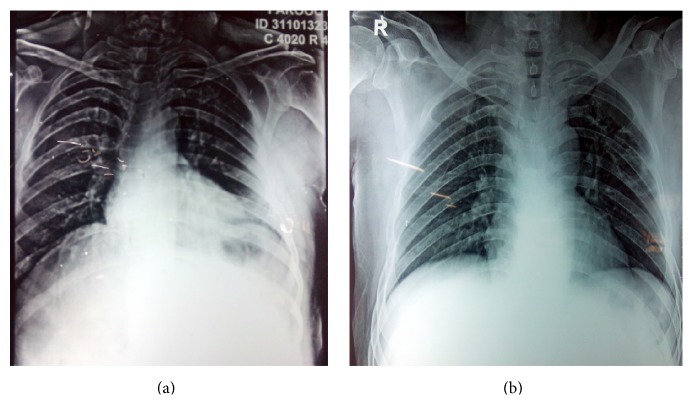
A chest X-ray on (a) showing left lower zone infiltrates. Chest X-ray on (b) at the time of discharge showing improvement.

**Figure 2:**
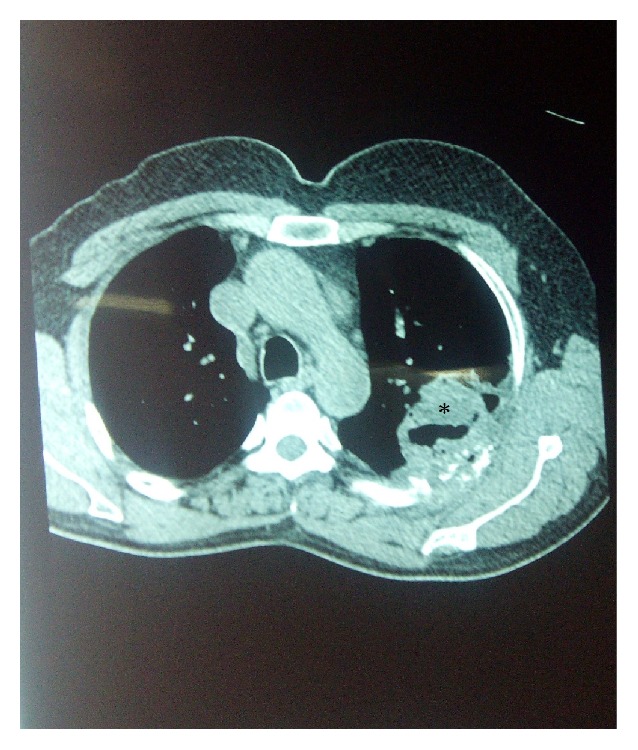
CECT chest showing the pulmonary cavity (pointed by the asterisk).

**Figure 3:**
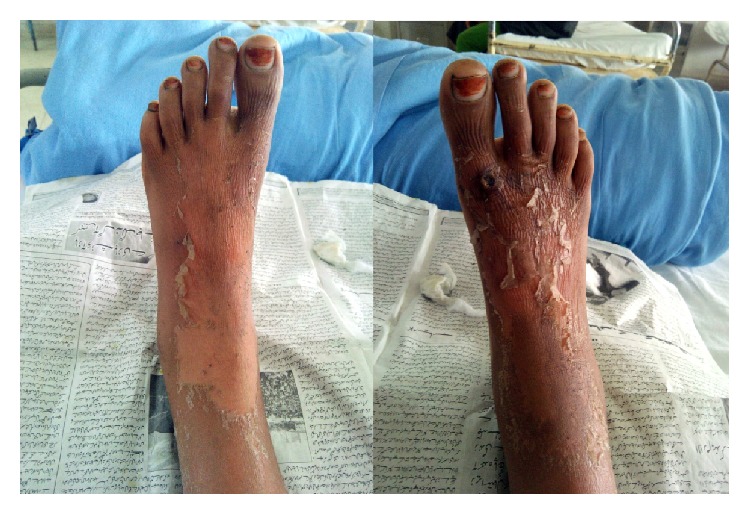
Desquamative rash on the feet of the patient.

The patient was a 55-year-old male who had undergone live related kidney transplantation 8 months prior. For induction the patient had received antithymocyte globulin (equine) at 15 mg/kg for 5 days. He was on maintenance immunosuppressive therapy with tacrolimus, mycophenolic acid mofetil, and prednisone. He presented with gradually worsening cough, expectoration, and fever for a couple of weeks. On examination he had crepitations in left lower lung fields posteriorly. Of particular note was poor orodental hygiene with multiple carious teeth. Chest roentgenogram showed infiltrates in left lower zone (Figure 1(a)). Other laboratory investigations were normal except for a mild neutrophilic leukocytosis. A CECT chest was done and a thick walled cavity was seen in the lower lobe of left lung posteriorly (Figure 2). While these evaluations were done patient continued to have fever and developed a desquamative rash on feet (Figure 3). A skin biopsy taken showed nonspecific inflammatory response and some epidermal necrosis; however, there was no evidence of any drug reaction or staphylococcal infection. The material obtained from skin biopsy did not grow any organism on culture. A blood culture was taken and patient was empirically given ceftriaxone injections. A CT guided transthoracic lung biopsy was done which showed nonspecific inflammatory response and no microorganism was seen on Gram and acid fast staining. There was no clinical or laboratory evidence of tuberculosis, fungal infections, malignancy, or vasculitis. Also there was no evidence of viral infections like HIV, CMV, or hepatitis virus infections. Blood culture and the material obtained from the lung biopsy material both grew a* Neisseria species*. The isolate was oxidase and catalase positive but superoxol negative. Genetic testing showed 99% homology with both* N. lactamica *and* Neisseria polysaccharea.* The* Neisseria* species isolated was* o*-nitrophenyl-*β*-D-galactopyranoside positive and generated acid from sucrose. This helped in excluding* N. polysaccharea*. The organism was thus identified both echocardiogram showed no endocarditis. The kidney functions were normal and there was no evidence echocardiogram showed no endocarditis. The kidney functions were normal and there was no evidence of graft rejection.

As patient had been on immunosuppressive drugs for a long time it was postulated that the lack of immune response in the patient had caused this normally commensal microorganism to become pathologically invasive causing septicemia, a lung cavity, and an unusual rash. The rash on the feet was strongly considered to be related to* Neisseria lactamica* infection as there is no evidence for any alternative cause for the rash. Patient received a 4-week course of ceftriaxone and two weeks of levofloxacin. He showed good response to the antibiotics and was discharged in a stable condition with a normal roentgenogram(Figure 1(b)).

## 3. Discussion

A cavity is defined pathologically as a gas-filled space within a zone of pulmonary consolidation or within a mass or nodule. Radiographically, it is a lucency within a zone of pulmonary consolidation, a mass, or a nodule [1]. Some of the important causes of lung cavities include lung malignancy, rheumatological diseases like Wegener's granulomatosis, and infections like tuberculosis, fungal infections, necrotizing pneumonias, lung abscesses, and septic pulmonary emboli [2]. Immunocompromised host is a patient who is at increased risk for life-threatening infections due to a congenital or acquired abnormality of the immune system [3]. Among the pulmonary complications that occur in this kind of patient population, infections are the most common. They account for about 75% of pulmonary complications and are associated with high morbidity and mortality [4]. Some studies have found a correlation between wall thicknesses of a cavity with etiology on plain radiography. Cavities with a maximum wall thickness of 4 mm or less are considered to be usually caused by nonmalignant processes. Cavities with a maximum wall thickness of 5 to 15 mm can be malignant or nonmalignant. Cavities with a maximum wall thickness of >15 mm are usually malignant [5, 6]. However, some studies done later show that wall thickness is an imperfect tool for discriminating between malignant and nonmalignant etiologies of lung cavities. The use of cavity wall thickness to differentiate among infectious etiologies of pulmonary cavities is even more controversial [7]. So, in such a scenario, the best diagnosis can come by obtaining a tissue sample as was done in our case.

*Neisseria lactamica* is a gram-negative diplococcus bacterium. It is a part of the commensal bacterial flora of the human upper respiratory tract and shares this ecological niche with* Neisseria meningitidis*.* N. lactamica* is naturally transformable and can transfer antibiotic resistance markers into the closely related species* N. meningitides* [8]. In normal humans it may even be beneficial as natural immunity to* Neisseria meningitidis* may result from nasopharyngeal carriage of closely related commensal* Neisseria lactamica* [9]. However, in immunocompromised hosts, it has been known to cause diseases rarely. We found one case of a cavitatory pulmonary disease in an immunocompromised patient caused by* Neisseria lactamica* [10].* This was the first ever case reported making our case the second. Apart from this there has been a case of N. lactamica* bacteremic pneumonia in an adult with liver cirrhosis reported in 2006, a 1991 report of* N. lactamica* meningitis following skull trauma and a 2010 case of* N. lactamica* arthritis and septicemia in a patient immunosuppressed by myeloma and corticosteroids [11–13].

## 4. Conclusion

With the rapidly spreading medical advance more and more patients are getting organ transplants, so the population of immunocompromised people is on the rise. Microbiology has advanced too and now we have more tools to definitively identify rare and less expected microorganisms. The identification of* Neisseria lactamica* in a lung cavity in our case is one such outcome of the scenario. We expect more sinister and less expected organisms to cause diseases in the population of patients who have organ transplants. Clinicians need to be aware of these possibilities for patient care.

## Abbreviations

CECT: Contrast enhanced computed tomography.
